# Transcriptome Reversal in Sulfate Transporter Involves Abiotic Stress in *Sesuvium portulacastrum* L.

**DOI:** 10.3390/biology15161416

**Published:** 2026-08-18

**Authors:** Yingyi Yu, Minghua Luo, Yan Leng, Xuwen Shen, Zijun Zhao, Changwei Zhou, Wei Li, Shugang Hui

**Affiliations:** 1 Key Laboratory of Plant Resources Conservation and Germplasm Innovation in Mountainous Region, Ministry of Education, Institute of Agro-Bioengineering, College of Life Sciences, Guizhou University, Guiyang 550025, China; yuyingyi050208@163.com (Y.Y.); lengyan2500110168@163.com (Y.L.); shenxuwen12365@163.com (X.S.); zhaozijun2705@163.com (Z.Z.); changwei.1981@163.com (C.Z.); liw@gzu.edu.cn (W.L.); 2 Rubber Research Center, Yunnan Dehong Institute of Tropical Agricultural Science, Ruili 678600, China

**Keywords:** *Sesuvium portulacastrum*, halophyte, *SpSULTR3;1*, *SpSULTR3;2*, salt stress, heavy metals stress

## Abstract

Sulfur is an essential nutrient involved in plant growth, redox regulation, and responses to environmental stresses. Sulfate transporters (SULTRs) control sulfate uptake and distribution, thereby affecting sulfur availability for metabolic processes and stress adaptation. However, the characteristics and stress-responsive functions of *SULTRs* in the halophyte *Sesuvium portulacastrum* remain unclear. In this study, we identified and characterized the *SULTR* family in *S. portulacastrum* through phylogenetic analysis, gene structure comparison, conserved motif analysis, promoter characterization, synteny analysis, and expression profiling. A total of 22 *SpSULTRs* were identified and classified into three subfamilies. Most SpSULTRs contained conserved Sulfate_transp and STAS domains and were predicted to localize to the plasma membrane. Transcriptome analysis combined with qRT-PCR validation revealed that *SpSULTRs* displayed diverse tissue-specific expression patterns under salt, cadmium, and copper stresses. Of these, *SpSULTR3;1* and *SpSULTR3;2* showed strong responses to salt stress and were mainly expressed in leaves. Protein interaction predictions suggested that these two transporters may be associated with sulfur assimilation, antioxidant metabolism, and stress-related pathways. These results reveal the structural diversification and stress-responsive characteristics of the *SULTR* family in *S. portulacastrum* and provide a basis for further investigation of sulfur transport mechanisms underlying halophyte adaptation.

## Simple Summary

Sulfur is an important nutrient that supports plant growth, metabolism, and responses to environmental stress. Plants require efficient sulfur transport systems to maintain sulfur balance, especially when exposed to saline soils or heavy metal contamination. However, the regulation of sulfur transport in halophytes that naturally tolerate extreme environments remains largely unknown. In this study, we investigated sulfate transporter (SULTR) genes in *Sesuvium portulacastrum*, a coastal plant with strong salt tolerance. We identified 22 *SpSULTRs* and found that these genes have diversified during evolution and display distinct expression patterns in different tissues. When exposed to salt, cadmium, and copper stresses, several *SpSULTRs* showed stress-responsive expression changes. Of these, *SpSULTR3;1* and *SpSULTR3;2* exhibited strong responses to salt treatment, suggesting that they may contribute to sulfur regulation during stress adaptation. Protein interaction analysis further indicated that these transporters may be connected with sulfur metabolism and stress-related pathways. This study improves our understanding of how sulfate transport systems function in halophytes and provides a foundation for future research on sulfur-mediated stress tolerance in plants.

## 1. Introduction

Soil salinization is a pervasive environmental constraint that limits plant growth and agricultural productivity across global ecosystems [1]. High salinity imposes simultaneous osmotic and ionic stresses on plants, reducing soil water potential and restricting water uptake, which results in physiological drought conditions even under sufficient soil moisture [2,3]. Excessive accumulation of Na^+^ and Cl^−^ disrupts intracellular ion homeostasis, inhibits enzyme activity, and interferes with core metabolic processes. These ionic imbalances also impair the uptake of essential nutrients such as potassium and calcium, thereby further restricting plant development. Salinity stress additionally triggers excessive production of reactive oxygen species, which damage lipids, proteins, and nucleic acids [4]. Plant survival under such conditions relies on coordinated regulatory systems that integrate ion transport, osmotic adjustment, antioxidant defense, and hormonal signaling. In addition to salinity, heavy metal pollution poses another serious threat to plant growth and ecosystem stability [5]. Cadmium is a highly toxic non-essential metal that can accumulate in plant tissues, disturb mineral nutrient balance, inhibit photosynthetic activity, and induce oxidative stress [6]. Copper, although required as an essential micronutrient for numerous enzymatic reactions, becomes toxic when accumulated at excessive levels by disrupting redox homeostasis and stimulating reactive oxygen species (ROS) production [7]. Plants respond to heavy metal stress through multiple protective mechanisms, including metal chelation, antioxidant activation, ion transport regulation, and metabolic adjustment. Sulfur-containing metabolites, particularly glutathione and phytochelatins, are important components of heavy metal detoxification because they participate in metal binding and oxidative stress protection [8]. Therefore, sulfur transport and assimilation may contribute to plant tolerance against both salinity and heavy metal stresses. However, whether *sulfate transporters* (*SULTR*) participate in the coordinated regulation of multiple environmental stresses in halophytes remains largely unknown.

Most cultivated crops are glycophytes and display limited tolerance to high salinity. In contrast, the halophyte *Sesuvium portulacastrum* L. maintains active growth under extreme saline environments through efficient ion compartmentalization, enhanced redox regulation, and metabolic flexibility [9,10]. Multi-omics analyses have revealed extensive transcriptional and proteomic remodeling in response to salt stress, involving pathways associated with ion transport, redox regulation, hormone signaling, and energy metabolism [11,12,13]. These responses involve pathways related to ion transport, redox homeostasis, hormone signaling, photosynthesis, and secondary metabolism [11,14,15,16]. Functional studies have identified diverse components contributing to salinity adaptation, including ion transporters, signaling kinases, aquaporins, antioxidant enzymes, transcriptional regulators, and germin-like proteins, which collectively sustain cellular homeostasis under stress conditions [13,17,18,19,20,21,22,23,24,25]. Despite these advances, how nutrient transport systems are integrated into the salinity adaptation network of halophytes remains poorly understood.

Sulfur metabolism is a central component of plant stress physiology. SULTRs control sulfur uptake, translocation, and intracellular distribution, thereby defining sulfur availability under environmental constraints. Sulfate assimilation provides cysteine, methionine, and glutathione, which are essential for redox homeostasis and detoxification processes [26,27]. In Arabidopsis, high-affinity sulfate transporters such as SULTR1;1 and SULTR1;2 regulate sulfate uptake in response to external sulfur status and developmental cues [28,29,30]. Enhanced sulfate assimilation promotes glutathione biosynthesis and alleviates oxidative injury under salinity stress [31]. Sulfur availability also modulates cysteine homeostasis, hormone signaling, and stress responses, including abscisic acid-mediated pathways [32]. In addition, sulfur nutrition improves tolerance to abiotic stresses by limiting toxic ion accumulation and strengthening antioxidant capacity [33]. Sulfate metabolism interacts with hormone signaling pathways, including abscisic acid, which regulates stress responses under saline conditions [34]. Regulatory networks involving miR395 further connect sulfur metabolism with environmental adaptation [35]. These findings position sulfate transporters as central regulators linking nutrient flux with stress adaptation. However, their evolutionary organization and stress-responsive functions in halophytes remain largely unexplored.

In this study, we propose that the *SULTR* family in *S. portulacastrum* has experienced functional diversification and that specific members may participate in environmental adaptation by regulating sulfate distribution and sulfur-related metabolic processes. To test this hypothesis, we performed a genome-wide identification and characterization of *SULTRs* in *S. portulacastrum*. The phylogenetic relationships, chromosomal distribution, gene structures, conserved motifs, promoter *cis*-elements, and duplication patterns of *SpSULTRs* were systematically analyzed. Transcriptome sequencing and quantitative reverse transcription polymerase chain reaction (qRT-PCR) assays were conducted to investigate the expression responses to salt, Cadmium, and Copper stress. Subcellular localization and protein–protein interaction analyses were performed to explore the potential functions and regulatory relationships of candidate SULTRs. This study provides new insights into the role of sulfate transport systems in halophyte stress adaptation and identifies potential genetic resources for improving crop stress tolerance.

## 2. Materials and Methods

### 2.1. Identification of SpSULTRs in the Genome of S. portulacastrum

The reference genome of *S. portulacastrum* was obtained from the Chinese National Genebank database under accession number CNP0008816 [13]. Protein sequences of sulfate transporters from *Arabidopsis thaliana* and *Oryza sativa* were obtained from the Arabidopsis Information Resource and the Rice Genome Annotation Project databases. These sequences were used as queries for homology searches against the *S. portulacastrum* proteome. Sequence similarity searches were performed in TBtools (v2.485) with an E-value threshold of 1 × 10^−5^. Candidate proteins were further screened using HMMER based on conserved Pfam domains PF00916 and PF01740 with an E-value cutoff of 1 × 10^−5^. All candidate sequences were examined to confirm the presence of conserved domains and to remove redundant entries.

### 2.2. Phylogenetic and Sequence Analysis

Protein sequences of SpSULTRs, AtSULTRs, and OsSULTRs were aligned, and a phylogenetic tree was constructed using MEGA11. The Neighbor-Joining method was applied with 1000 bootstrap replicates and the K80 substitution model, following the procedure described previously [36]. Subcellular localization was predicted using the WoLF PSORT online tool. Physicochemical parameters, including molecular weight and isoelectric point, were calculated using the ProtParam server (https://npsa.lyon.inserm.fr/cgi-bin/npsa_automat.pl?page=/NPSA/npsa_sopma.html, accessed on 13 August 2026) as described previously [21]. Conserved motifs were identified using the MEME (v5.5.9) suite under default parameters.

### 2.3. Promoter Analysis of Cis-Elements

Promoter regions of 2000 base pairs upstream of the start codon of each *SpSULTR* gene were extracted from the genome. *Cis*-elements within these sequences were identified using the PLANTCARE database. The distribution of *cis*-elements was visualized using the Simple BioSequence Viewer module in TBtools (v2.485) [25]. A heat map representing the abundance and classification of *cis*-elements was generated using the HeatMap function in TBtools (v2.485).

### 2.4. Collinearity Analysis of SpSULTRs

Collinearity analysis within the *S. portulacastrum* genome and between *S. portulacastrum*, *A. thaliana*, and *O. sativa* was conducted using TBtools (v2.485). Input files for MCScanX were prepared with the File Merger module. The E-value threshold for homologous gene detection was set to 1 × 10^−10^. Syntenic relationships were visualized using the Circle Gene Viewer function in TBtools (v2.485).

### 2.5. Protein–Protein Interaction Prediction

Potential interactions among SpSULTRs were predicted using the STRING database. Full-length amino acid sequences were used as queries and mapped to the *A. thaliana* database because no direct interaction information is available for *S. portulacastrum*. The protein sequences of SpSULTR3;1 and SpSULTR3;2 were used as query sequences to identify putative interacting partners. Interaction data were generated from multiple sources in STRING, including co-expression, gene neighborhood, experimental evidence, and literature mining. Only interactions that passed a medium-to-high-confidence cutoff were retained for further analysis. The interaction network was visualized in Cytoscape (version 3.9.1). Network topology was used to identify highly connected nodes and potential interaction modules. The resulting interaction networks were imported into Cytoscape (version 3.10.1) for visualization and network analyses. Candidate interacting proteins were subjected to Gene Ontology (GO) and Kyoto Encyclopedia of Genes and Genomes (KEGG) enrichment analyses to identify biological processes and metabolic pathways associated with SpSULTR3;1 and SpSULTR3;2. Significantly enriched GO terms were classified into biological processes, molecular functions, and cellular components. KEGG pathways related to sulfur metabolism, glutathione metabolism, photosynthesis, and stress responses were further evaluated. The enrichment significance was determined using FDR correction, and pathways with FDR < 0.05 were considered significantly enriched.

### 2.6. Chromosomal Position Analysis of SpSULTRs

The chromosomal distribution of *SpSULTRs* was analyzed using TBtools (v2.485). Genome annotation data and gene lists were imported into the software, and genes were mapped according to their physical positions on chromosomes. The resulting map was adjusted to improve clarity and visual presentation.

### 2.7. Structure and Evolutionary Analysis

Secondary structures of SpSULTRs were predicted using SOPMA (https://npsa.lyon.inserm.fr/cgi-bin/npsa_automat.pl?page=/NPSA/npsa_sopma.html, accessed on 13 August 2026). Three-dimensional structures were modeled using SWISS-MODEL (https://swissmodel.expasy.org/) as previously described [25]. Full-length amino acid sequences of SpSULTRs were used as inputs for homology modeling. Each sequence was submitted to the SWISS-MODEL (https://swissmodel.expasy.org/) workspace for template identification, and suitable structural templates were selected based on sequence similarity, sequence coverage, and quality of the available experimental structures. The selected templates were used to construct three-dimensional models using the SWISS-MODEL (https://swissmodel.expasy.org/) homology modeling pipeline. The quality of each predicted model was assessed using the Global Model Quality Estimation and QMEAN scores provided by SWISS-MODEL (https://swissmodel.expasy.org/). The GMQE score ranges from 0 to 1, with higher values indicating greater expected accuracy of the model. The QMEAN score was used as an additional measure of overall model quality, with values closer to 0 indicating better agreement with expected structural features. The model with suitable template coverage and the highest overall quality was retained for subsequent structural analysis. The predicted structures were examined to assess their overall conformations and conserved structural features among the SpSULTRs.

Gene duplication events were identified through collinearity analysis, and duplication types were classified based on their genomic relationships. Nonsynonymous and synonymous substitution rates were calculated using TBtools (v2.485) to evaluate selective pressure among duplicated gene pairs.

### 2.8. Transcriptome Analysis of the Expression of SpSULTRs

Plant materials of *S. portulacastrum* were collected from Hainan Province, China. Stem cuttings (approximately 3 cm long) were excised from the upper region of mature plants. Each cutting contained one node and two leaves. The cuttings were cultivated in 1/2 Hoagland nutrient solution supplemented with 400 mM NaCl under controlled conditions with a 12 h light/12 h dark photoperiod at 28 °C in a greenhouse. The transcriptome dataset generated under copper stress was obtained from our previous study [37]. Briefly, *S. portulacastrum* plants were cultured hydroponically with different concentrations of Cu(NO_3_)_2_ (0, 50, 100, and 200 µM), and transcriptome sampling and analysis were performed. Transcriptomic data under cadmium and salt stress were obtained using the same experimental framework. For cadmium treatment, plants were exposed to 200 µM CdCl_2_, and samples were collected at 0, 7, 14, and 21 days after treatment. Salt stress experiments were conducted using NaCl concentrations ranging from 0 to 1000 mM (0, 200, 400, 600, 800, and 1000 mM) according to a previously reported protocol [13]. Transcriptome sequencing, data processing, and expression analyses were performed as previously described [13,37]. The changes in the expression of genes responding to cadmium stress are listed in Table S1.

### 2.9. Quantitative Real-Time PCR Analysis

For the time-course experiment under salt stress, *S. portulacastrum* seedlings were exposed to 0.6 M NaCl solution. Leaf and root tissues were sampled at 0, 2, 8, 12, and 24 h after treatment. Samples collected before NaCl exposure were used as the 0 h control. At each time point, leaves and roots were harvested separately, immediately frozen in liquid nitrogen, and stored at −80 °C until RNA extraction. Each treatment included at least three independent biological replicates.

Total RNA was extracted from leaf and root tissues using a plant RNA extraction kit R401 (Vazyme, Nanjing, China) following the manufacturer’s instructions. RNA quality and concentration were checked by agarose gel electrophoresis and spectrophotometry, and only intact RNA samples were used for downstream experiments. First-strand cDNA was synthesized from 1 μg of total RNA using a reverse transcription kit R021 (Vazyme, Nanjing, China). Quantitative real-time PCR was performed on a qTOWER3G real-time PCR system (Analytik Jena, Germany) with SYBR Green chemistry. Primers for *SpSULTRs* were designed based on conserved coding sequences, and primer specificity was verified before use (Table S2). The transcript levels of *SpGAPDH* served to normalize the expression levels of the target genes [21,25]. Each reaction contained SYBR Green master mix, diluted cDNA, and gene-specific primers in a final volume of 20 μL. The amplification program followed a standard cycling procedure with denaturation, annealing, and extension steps. Each sample was analyzed with three biological replicates and three technical replicates. Gene expression levels were normalized to an internal reference gene and used for relative quantification.

### 2.10. Subcellular Localization

Full-length coding sequences of *SpSULTR3;1* and *SpSULTR3;2* were fused with GFP and transiently expressed in *Nicotiana benthamiana* leaves. A plasma membrane marker, OsSULTR2;1 fused with mCherry, was co-expressed for localization reference [35]. Fluorescence signals were observed using a Leica TCS SP8 confocal microscope.

## 3. Results

### 3.1. Identification and Characterization of SpSULTRs

A total of 22 *SpSULTRs* were identified in the genome of *S. portulacastrum* through HMM-based screening and sequence similarity searches (Table S3). Based on their phylogenetic relationships, these genes were designated as *SpSULTR1;1* to *SpSULTR4;3*. The predicted protein lengths ranged from 217 amino acids in SpSULTR1;4 to 698 amino acids in SpSULTR4;1 and SpSULTR4;2. The molecular weights varied from 24.52 kDa for SpSULTR1;4 to 76.06 kDa for SpSULTR4;1. The predicted isoelectric points ranged from 5.56 in SpSULTR3;1 to 9.24 in SpSULTR3;9, with most members exhibiting alkaline pI values above 8.0.

Subcellular localization prediction indicated that all SpSULTRs are localized to the plasma membrane (Table S3). Secondary structure analysis revealed that all members consisted mainly of α-helices, extended strands, and random coils (Table S4). The proportion of α-helices ranged from 46.85% in SpSULTR4;2 to 53.57% in SpSULTR3;12. Extended strands accounted for 9.15% to 15.21% of the protein structures, whereas random coils represented 33.64% to 44.93% of the total structure. Three-dimensional modeling revealed a conserved spatial structure throughout the family (Figure S2). These results indicate a generally conserved structural organization among SpSULTRs.

### 3.2. Phylogenetic Analysis of the SpSULTRs

**Figure 1:**
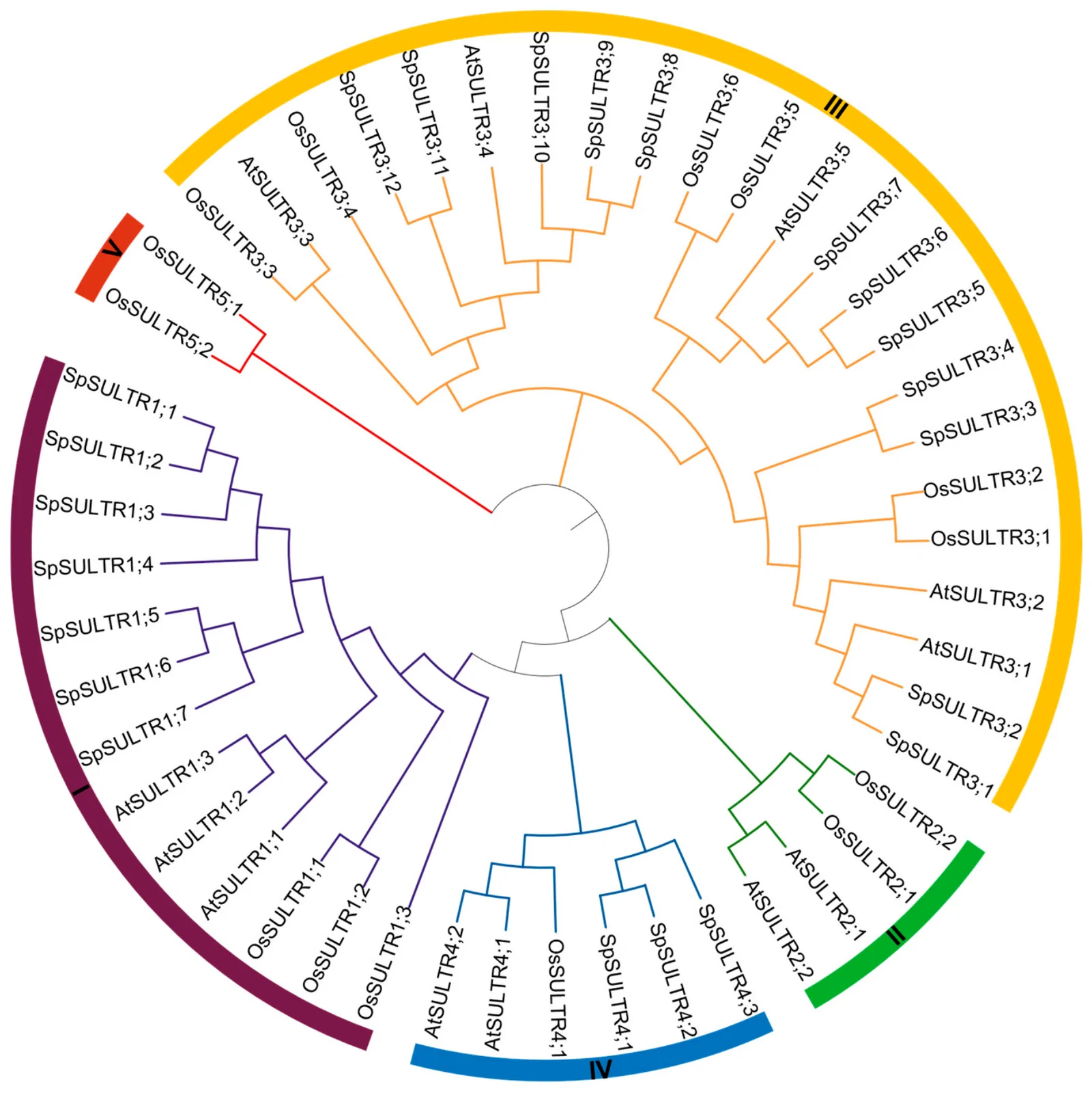
Phylogenetic analysis of SULTRs in *S. portulacastrum*, *A. thaliana* and *O. sativa*. The phylogenetic tree was constructed via the Neighbor-Joining method with 1000 bootstrap replicates using MEGA 11.

To investigate the evolutionary relationships of sulfate transporters in *S. portulacastrum*, a phylogenetic tree was constructed using the conserved SULTR sequences. Based on phylogenetic clustering, the 22 SpSULTRs were classified into three groups (Figure 1). Group I contained seven members designated SpSULTR1;1 to SpSULTR1;7. Group III comprised twelve members designated SpSULTR3;1 to SpSULTR3;12. Group IV included three members designated SpSULTR4;1 to SpSULTR4;3. No member was assigned to Group II. The uneven distribution of family members among different groups suggests lineage-specific expansion within the SpSULTR family.

### 3.3. Conserved Motifs and Gene Structure Analysis of SpSULTRs

**Figure 2:**
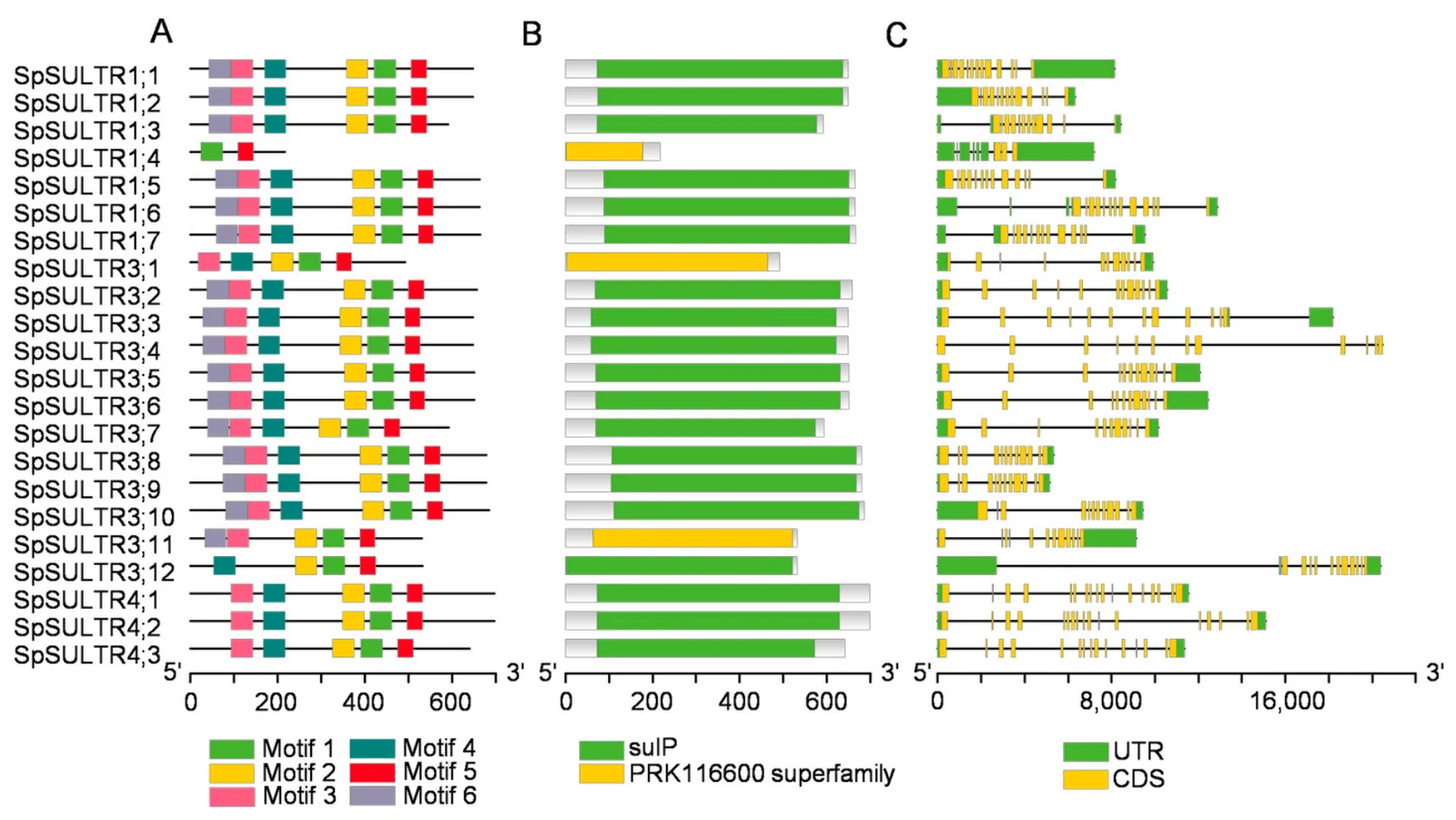
Gene and protein structure of SpSULTRs. (**A**) Motifs in the detected 22 SpSULTRs. (**B**) Conserved structural domains in the 21 SpSULTRs. (**C**) Gene structure analysis of the 22 *SpSULTRs*. The horizontal coordinate represents the length of the gene/amino acid sequence.

To examine structural conservation among SpSULTRs, six conserved motifs were identified and designated as Motif 1 to Motif 6 (Figure S1). Members within the same phylogenetic group exhibited highly similar motif compositions, indicating functional conservation among closely related proteins. Most SpSULTRs contained all six motifs, accounting for approximately 70% of the family members. Motifs 1 to 5 were present in all identified SpSULTRs, suggesting that these motifs represent core structural features of sulfate transporters. In contrast, SpSULTR1;4 contained the fewest motifs, implying a greater degree of functional divergence from other members (Figure 2A). Domain analysis showed that all SpSULTRs contained either the N-terminal Sulfate_transp domain or the C-terminal STAS domain (Figure 2B). Gene structure analysis revealed substantial variation in exon–intron organization among family members (Figure 2C). The number of exons ranged from three in SpSULTR1;4 to seventeen in SpSULTR4;1 and SpSULTR4;2. The number of introns varied from zero in SpSULTR3;4 to eight in SpSULTR1;4. These differences in protein and gene architecture indicate structural diversification during the evolution of the SpSULTR family.

### 3.4. Analysis of Cis-Elements in SpSULTR Promoters

**Figure 3:**
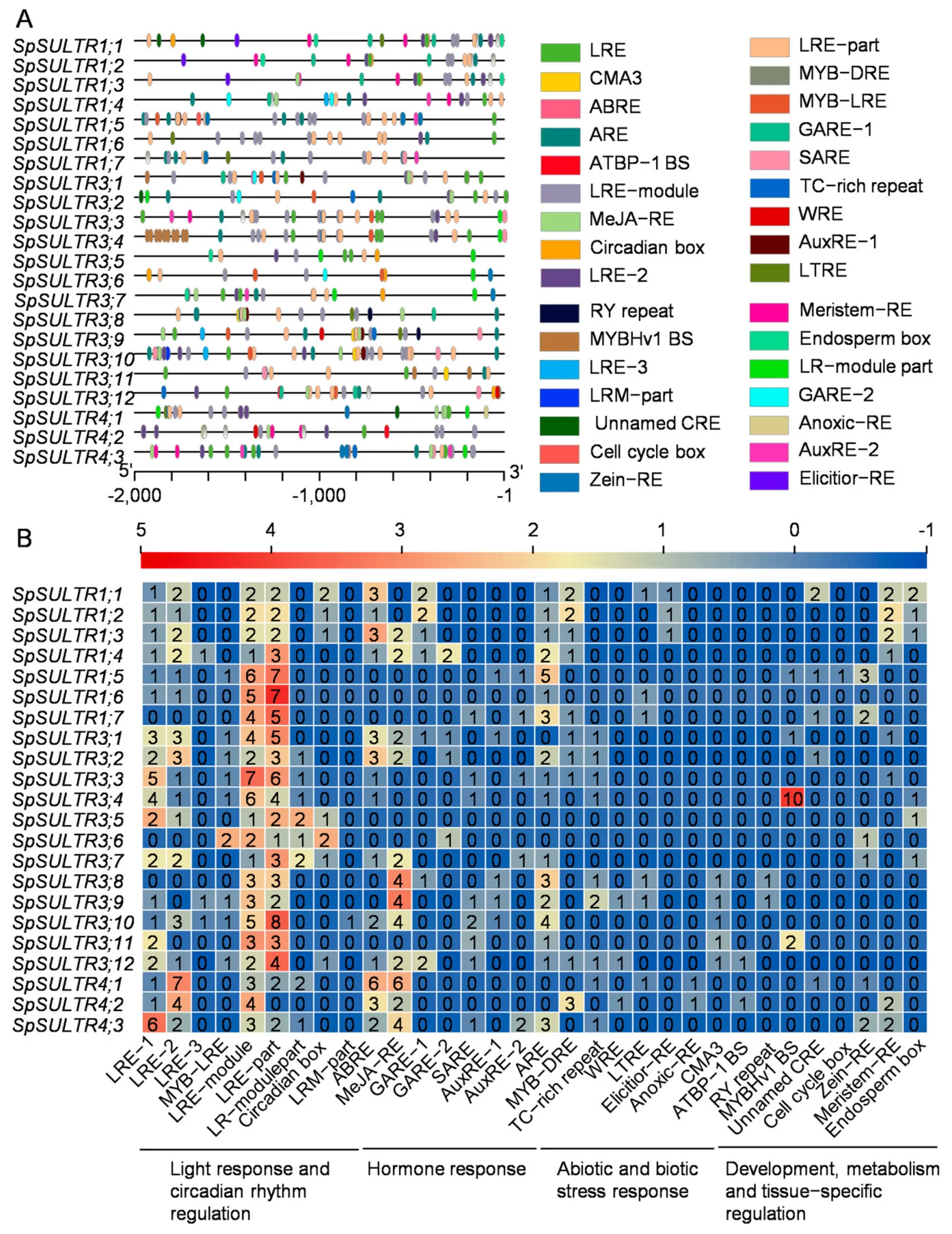
Predicted *cis*-elements in the promoter of *SpSULTRs*. (**A**). Category of *cis*-elements in the *SpSULTR* promoter. (**B**). Number of *cis*-elements in the *SpSULTR* promoter.

To explore the potential regulatory mechanisms of *SpSULTRs*, the 2 kb promoter regions upstream of each coding sequence were analyzed for *cis*-elements. A total of 32 *cis*-elements were identified and categorized into light-responsive and circadian rhythm-related elements, hormone-responsive elements, biotic and abiotic stress-responsive elements, and development-, metabolism-, and tissue-specific regulatory elements (Figure 3A). Among these categories, light-responsive and circadian rhythm-related elements were the most abundant and widely distributed across the *SpSULTR* promoters. MYBHv1BS and LRE-Part were among the most frequently detected elements. In contrast, *cis*-elements associated with hormone signaling, stress responses, development, metabolism, and tissue-specific regulation were present only in a subset of genes (Figure 3B). The differential distribution of these regulatory motifs suggests potential functional specialization among individual *SpSULTR* members and indicates that distinct genes may participate in different physiological and environmental response pathways.

### 3.5. Chromosomal Localization of SpSULTRs

**Figure 4:**
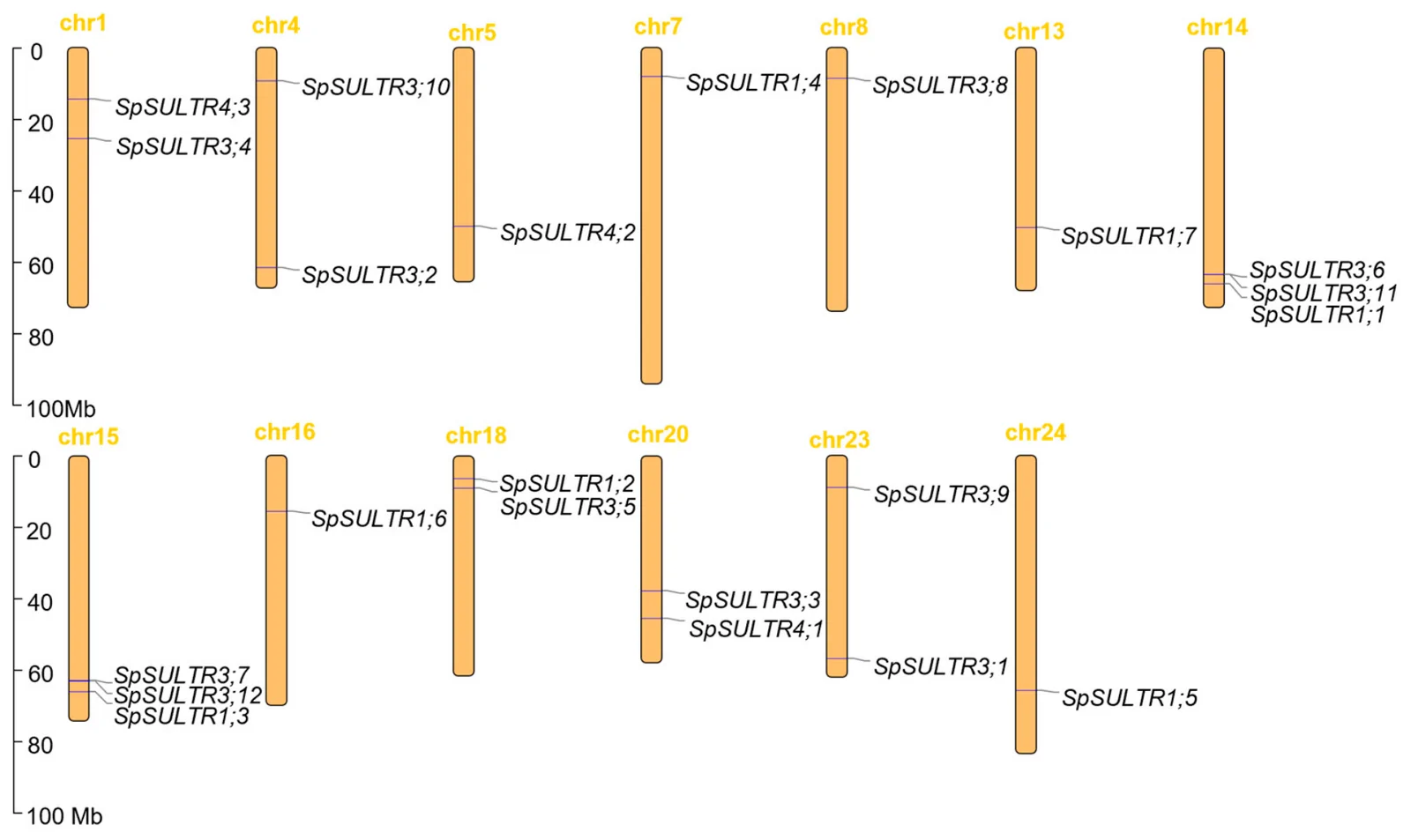
Chromosome distribution of *SpSULTRs* in *S. portulacastrum*.

The 22 *SpSULTRs* were unevenly distributed across 14 chromosomes of *S. portulacastrum* (Figure 4). Chromosomes 14 and 15 harbored the largest number of *SpSULTRs.* Chromosomes 1, 4, 18, 20, and 23 each contained two family members, whereas chromosomes 5, 7, 8, 13, 16, and 24 each carried a single *SpSULTR*. Most genes were located near the terminal regions of chromosomes, indicating a non-random chromosomal distribution pattern within the genome.

### 3.6. Collinearity Analysis of the SpSULTRs

**Figure 5:**
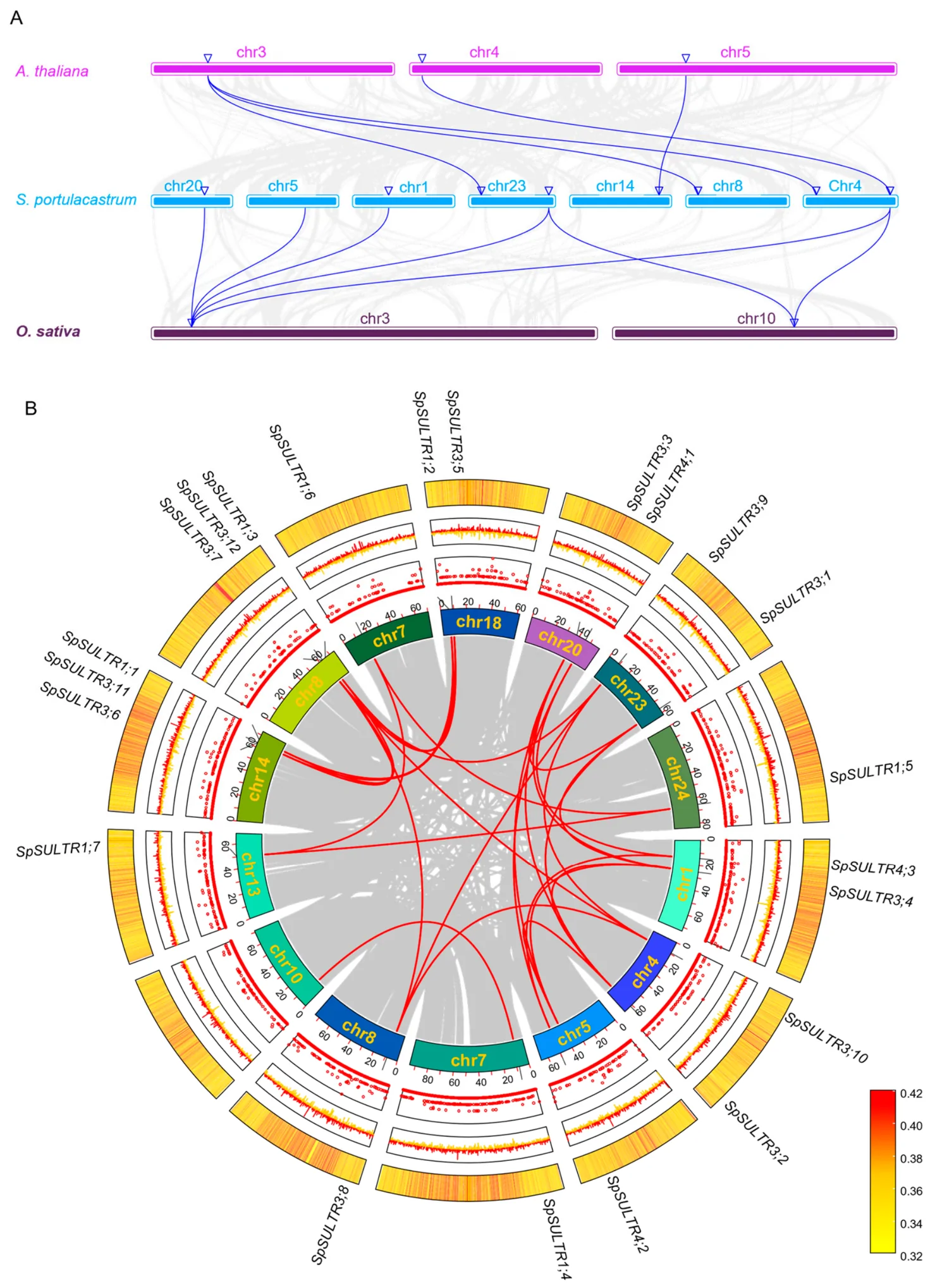
Collinearity analysis of SULTRs in *S. portulacastrum*. (**A**) Synteny analysis of SULTRs in *S. portulacastrum, A. thaliana and O. sativa*. (**B**) Collinearity analysis of SULTRs between *S. portulacastrum*.

Comparative synteny analysis identified four orthologous gene pairs between *S. portulacastrum* and *A. thaliana* and seven orthologous gene pairs between *S. portulacastrum* and rice (Figure 5A). These conserved collinear relationships indicate that several *SpSULTRs* share a common evolutionary origin with sulfate transporters from other plant species.

Within the *S. portulacastrum* genome, 25 collinear gene pairs were identified among the *SpSULTRs* (Figure 5B). Both intra-subgroup and inter-subgroup duplication events were detected. The Ka values of duplicated gene pairs ranged from 0.00339 (*SpSULTR3;4* and *SpSULTR3;3*) to 0.27954 (*SpSULTR3;4* and *SpSULTR3;1*), while Ks values ranged from 0.02998 (*SpSULTR4;3* and *SpSULTR4;1*) to 2.080 (*SpSULTR3;3* and *SpSULTR3;2*). All Ka/Ks ratios were lower than 0.4 (Table S5). Further analysis revealed that all duplicated *SpSULTRs* originated from either whole-genome duplication or segmental duplication events (Table S6). These duplication processes likely contributed to the expansion of the *SpSULTR* family and promoted functional diversification while maintaining the conserved functions of most duplicated genes.

### 3.7. Protein–Protein Interaction Analysis of SpSULTRs

**Figure 6:**
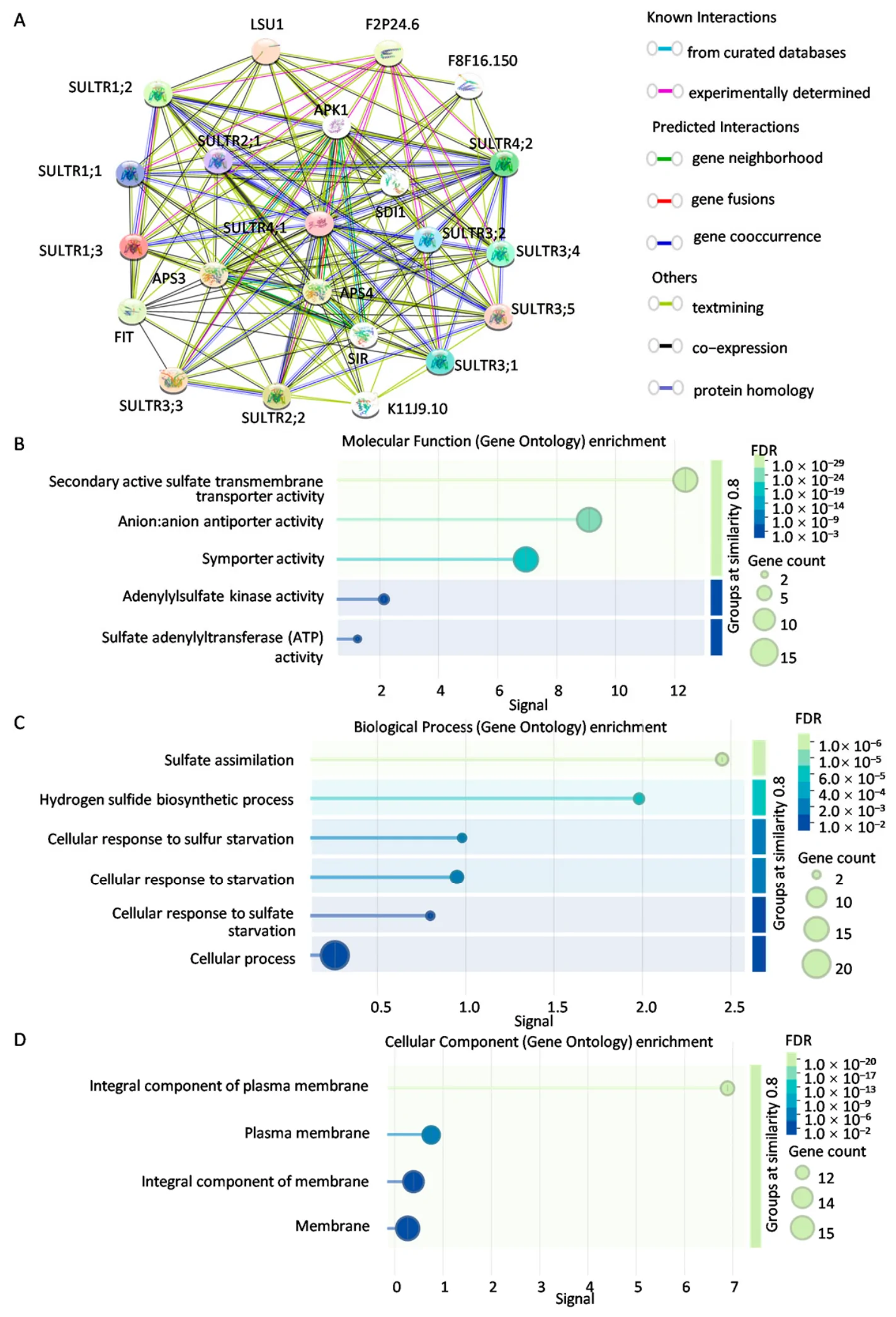
Predicted protein–protein interactions in SpSULTRs. (**A**) Interaction network of SULTRs. (**B**) Molecular function enrichment presented in the network. (**C**) Biological process enrichment presented in the network. (**D**) Cellular component enrichment presented in the network.

To gain insights into the potential functions of SpSULTRs, a protein interaction network was predicted using *A. thaliana* orthologs (Figure 6A). Most SpSULTRs were connected to one another and to enzymes involved in sulfate assimilation, including ATP sulfurylases (APS3 and APS4), sulfite reductase (SiR), and the sulfur deficiency-induced protein SDI1. The network places sulfate transporters within a broader sulfur metabolic context rather than as isolated transport components. SpSULTR3;1 and SpSULTR3;2 emerged as two of the most connected members of this family (Figure S3A,B). SpSULTR3;1 was linked to proteins associated with sulfur metabolism, particularly SiR and DES1, and proteins involved in chloroplast function and membrane transport, including TIC20-II and PHT2;1. Predicted associations with LHCB5 and ATL27 were also observed, suggesting that SpSULTR3;1 may be functionally related to chloroplast activity and stress-responsive processes. The interaction profile of SpSULTR3;2 was different. In addition to proteins involved in sulfur metabolism, such as APRL4, GSTU6, MOT1, and MOT2, it was associated with several components of the photosynthetic machinery, including LHCB5 and photosystem II proteins PsbD, PsbE, and PsbJ. The presence of GSTU6 within this network is notable because glutathione metabolism is a major sink for reduced sulfur during environmental stress.

The enrichment analysis was consistent with the network structure. Gene Ontology terms were dominated by sulfate assimilation, sulfur compound metabolism, sulfur biosynthetic processes, sulfate transport, sulfate reduction, and response to sulfur starvation. At the molecular function level, sulfate transporter activity, sulfate transmembrane transporter activity, ATP sulfurylase activity, sulfur compound binding, and oxidoreductase activity were significantly overrepresented (Figure 6B). Most of the corresponding proteins were predicted to localize to the plasma membrane, chloroplast, plastid envelope, and thylakoid membranes (Figure 6C). KEGG analysis highlighted sulfur metabolism as the most significantly enriched pathway. Glutathione metabolism, photosynthesis, photosynthetic antenna proteins, carbon fixation, and ABC transporters were also enriched, indicating that sulfur transport is closely associated with both primary metabolism and stress-related cellular processes (Figure 6D).

### 3.8. Expression Profile of SpSULTRs Under Salt Stress, Cadmium, and Copper

**Figure 7:**
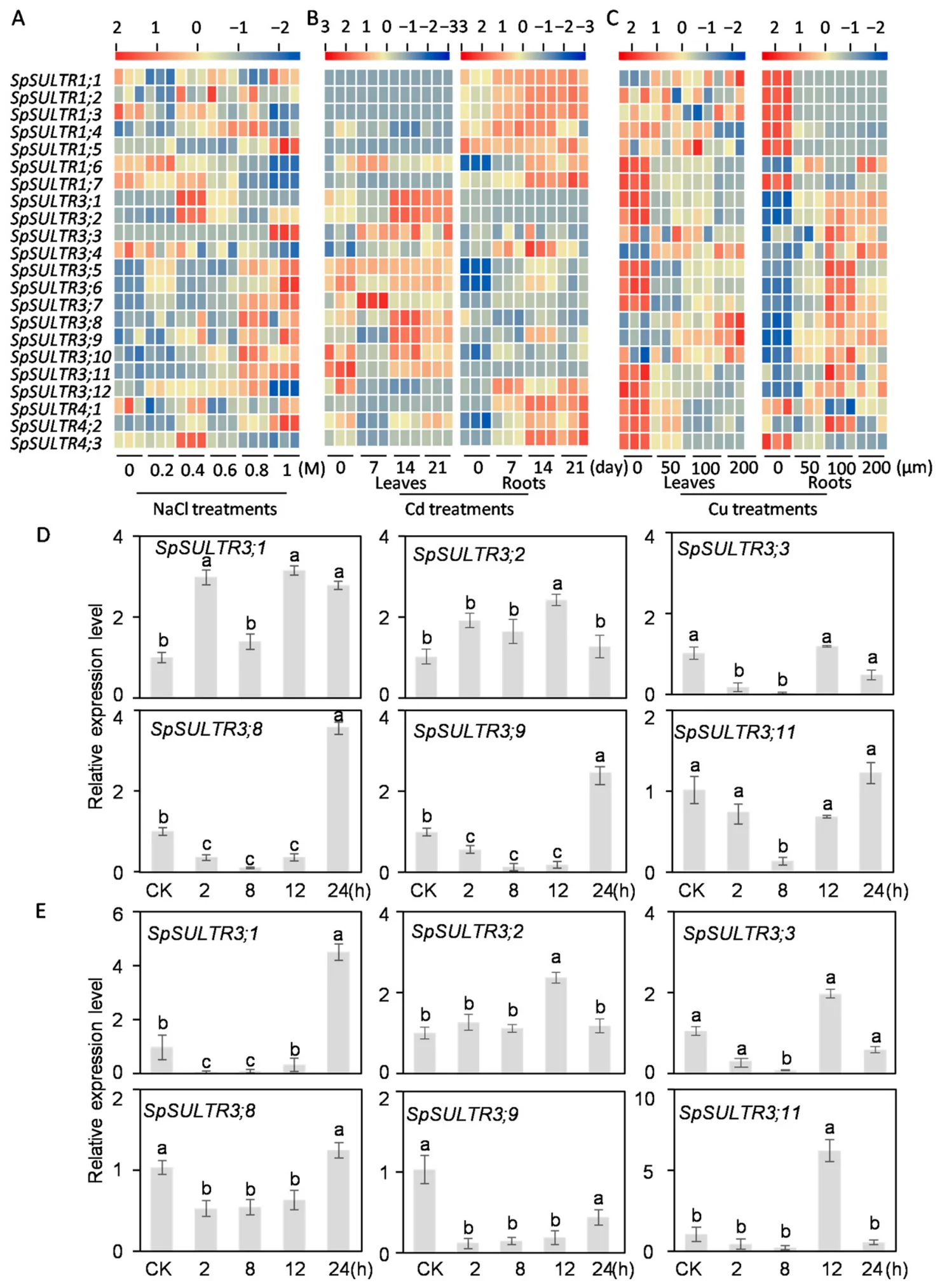
Expression patterns of *SpSULTRs* in *S. portulacastrum* under various abiotic stresses. (**A**) Fold change in *SpSULTRs* under salt stress. (**B**) Fold change in *SpSULTRs* under Cd stress. (**C**) Fold change in *SpSULTRs* under Cu stress. (**D**) qRT-PCR assay of the expression of *SpSULTRs* under salt treatment at different time points. Data represents mean ± SD (*n* = 3). The different letters above each bar indicate significant differences at *p* < 0.05 determined by one-way ANOVA analysis followed by Tukey’s multiple test. (**E**) qRT-PCR assay of the expression of *SpSULTRs* under salt treatment at different time points. Data represents mean ± SD (*n* = 3). The different letters above each bar indicate significant differences at *p* < 0.05 determined by one-way ANOVA analysis followed by Tukey’s multiple test.

To get a sense of what these *SpSULTRs* might actually be doing under stress, we pulled in the transcriptome data from salt, cadmium, and copper treatments and just looked at how messy or coordinated their expression gets. Under salt stress, the first thing that stands out is that not all genes behave the same at all. Some of them slowly drop as NaCl goes up, like *SpSULTR1;6*, *SpSULTR1;7*, and *SpSULTR3;4*. Others seem to peak at a middle point, around 0.4 M NaCl, including *SpSULTR3;1*, *SpSULTR3;2*, and *SpSULTR4;3*. Then there is another group that just keeps climbing with salinity, such as *SpSULTR1;5* and several *SpSULTR3* members including *SpSULTR3;3*, *SpSULTR3;6*, *SpSULTR3;7*, *SpSULTR3;8*, *SpSULTR3;9*, *SpSULTR3;11*, and *SpSULTR4;2* (Figure 7A). It does not look uniform at all, more like each gene is reacting in its own way depending on the level of stress. Cadmium stress is even more dynamic. In leaves, a few genes already sit at a relatively high baseline before treatment, like *SpSULTR3;5*, *SpSULTR3;6*, *SpSULTR3;10*, *SpSULTR3;11*, and *SpSULTR3;12*. After exposure, some genes respond later, not immediately. *SpSULTR3;3* and *SpSULTR3;7* start going up around day 7. By day 14, things become more obvious for *SpSULTR3;1*, *SpSULTR3;2*, and *SpSULTR3;9*, and by day 21 these three reach their highest levels. Roots look different again. Genes like *SpSULTR1;1*, *SpSULTR1;4*, *SpSULTR1;5*, *SpSULTR4;2*, and *SpSULTR4;3* already show high expression even before stress, and then they get pushed further upward during cadmium exposure (Figure 7B). Copper stress adds another layer of variation. In leaves, quite a few genes start off already active, including *SpSULTR1;6*, *SpSULTR1;7*, *SpSULTR3;1*, *SpSULTR3;2*, *SpSULTR3;5*, *SpSULTR3;6*, *SpSULTR3;7*, *SpSULTR3;11*, *SpSULTR3;12*, *SpSULTR4;1*, *SpSULTR4;2*, and *SpSULTR4;3*. After copper treatment, some genes clearly ramp up, especially *SpSULTR1;1*, *SpSULTR1;5*, *SpSULTR3;4*, *SpSULTR3;8*, and *SpSULTR3;9*. *SpSULTR1;5* in particular hits its highest level. At the same time, a few genes just keep going down in leaves, like *SpSULTR1;6*, *SpSULTR1;7*, and *SpSULTR3;1*. Roots show their own pattern too, where several genes including *SpSULTR1;1*, *SpSULTR1;2*, *SpSULTR1;3*, *SpSULTR1;5*, and *SpSULTR4;3* are gradually suppressed, while *SpSULTR4;2* only spikes briefly and then drops back (Figure 7C).

We also looked at a longer NaCl treatment at 0.6 M across different time points. Here, *SpSULTR3;1* and *SpSULTR3;2* clearly go up in leaves (Figure 7D), while *SpSULTR3;9* goes down in both leaves and roots (Figure 7D,E). The rest do not really follow one clean pattern; they shift depending on time and tissue (Figures S4 and S5). Putting everything together, *SpSULTR3;1* and *SpSULTR3;2* keep showing up as consistently responsive under salt stress, so they are probably more closely tied to salt adaptation than others in the family.

### 3.9. Subcellular Localization of SpSULTRs

**Figure 8:**
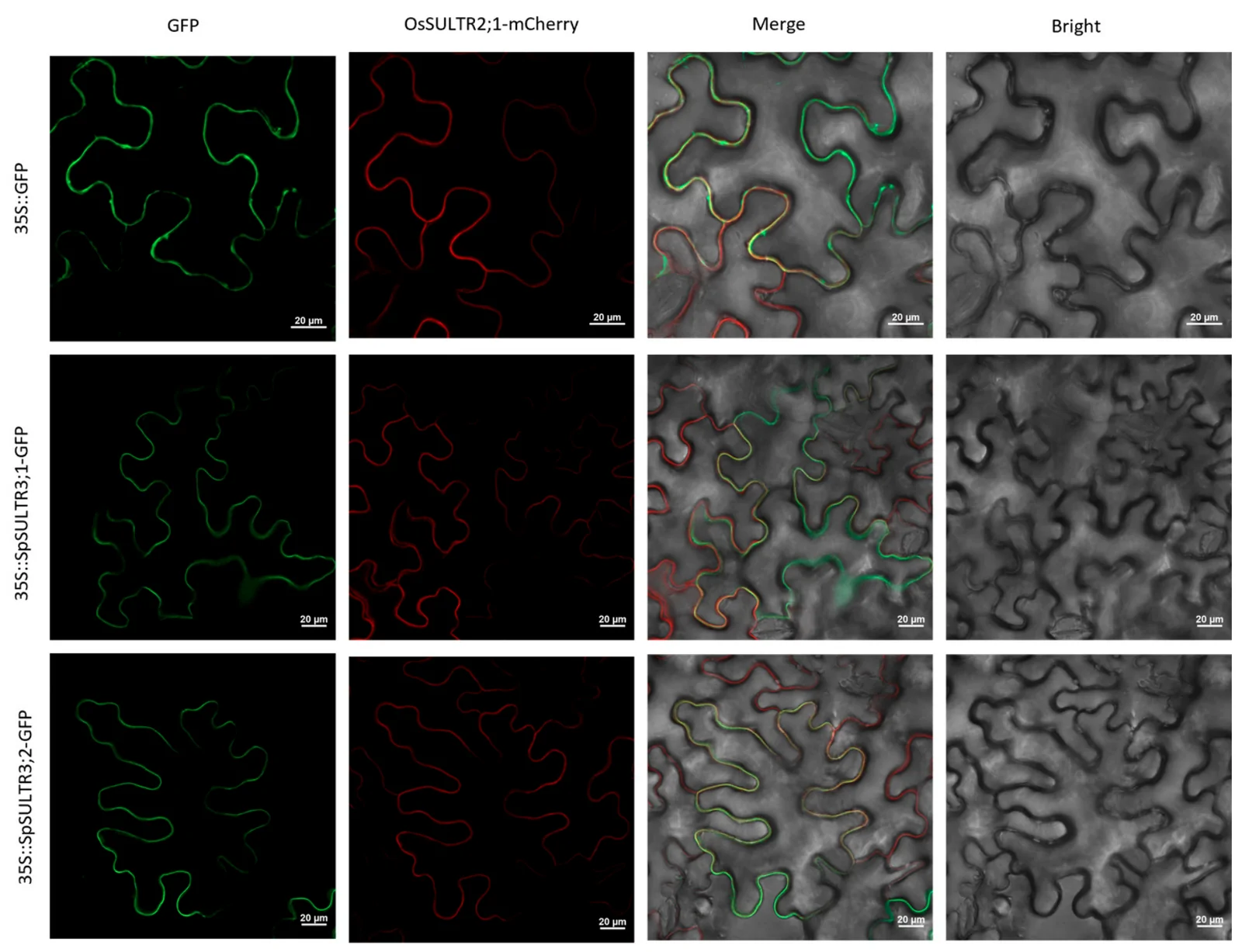
Subcellular localization of SpSULTRs of *S. portulacastrum* in tobacco leaves. Scale bars = 20 μm.

Subcellular localization prediction indicated that all SpSULTRs are targeted to the plasma membrane. To experimentally verify this prediction, SpSULTR-GFP fusion proteins were co-expressed with the plasma membrane marker OsSULTR2;1-mCherry in *Nicotiana benthamiana* leaf epidermal cells. Confocal microscopy revealed that the GFP fluorescence signals overlapped with the mCherry signals at the cell membrane (Figure 8). The co-localization of both fluorescence signals confirmed that SpSULTR3;1 and SpSULTR3;2 are localized to the plasma membrane. This localization pattern is consistent with their proposed roles in sulfate uptake and transmembrane transport.

## 4. Discussion

Sulfur metabolism is essential for plant growth and environmental adaptation, and sulfate transporters constitute the primary gateway that links external sulfur availability with intracellular metabolic requirements [38,39,40,41,42]. Previous studies have identified SULTR family members in numerous plant species, including *A*. *thaliana* and *O*. *sativa* [43,44]. In this study, 22 *SpSULTRs* were identified in the halophyte *S. portulacastrum*. These genes exhibit conserved structural features, including Sulfate_transp and STAS domains, and most proteins are localized to the plasma membrane. Promoter analysis revealed cis-elements associated with light, hormone, and stress responses, indicating that SULTRs integrate nutrient acquisition with environmental and developmental regulations.

A notable feature of the *S. portulacastrum* SULTR family is the absence of the SULTR2 subfamily, which in Arabidopsis mediates sulfate redistribution between roots, shoots, and reproductive tissues [38,45,46,47]. This pattern suggests a distinct organization of sulfate transport in halophytic species. The absence of SULTR2 members may reflect evolutionary reconfiguration of systemic sulfur allocation rather than simple gene loss. In saline coastal environments, external sulfate availability and strong ionic flux may reduce dependence on long-distance sulfate transport systems, which may relax selective constraints on SULTR2 maintenance. Under these conditions, sulfate distribution may rely more on localized transport and tissue-specific regulation.

The expansion of SULTR1 and SULTR3 members supports a compensatory shift in sulfate transport capacity toward high-affinity uptake and intracellular redistribution. This pattern indicates a transition from centralized transport control to a distributed system that maintains sulfur homeostasis through redundancy and local regulation. Similar gene family expansions and contractions have been observed in transporter systems during plant adaptation to specific ecological niches, supporting the role of gene family plasticity in environmental adaptation [48].

Sulfur assimilation is closely linked to redox regulation through the production of cysteine, methionine, and glutathione [49,50]. These compounds function in reactive oxygen species detoxification and metal chelation [51,52,53,54]. The responsiveness of *SpSULTRs* to salt, cadmium, and copper stresses indicates that sulfate transport is integrated into multiple stress response pathways. These stresses converge on a shared requirement for sulfur-containing metabolites, which suggests that SULTR regulation reflects a unified metabolic response to changes in cellular redox and detoxification demand.

Salt-stress-induced coordinated expression changes in multiple *SpSULTRs*, suggesting increased sulfate flux to support osmotic adjustment and redox balance. This pattern is consistent with a mechanism in which sulfur assimilation functions as a buffering system that stabilizes cellular homeostasis under ionic stress [49,54]. In halophytes such as *S. portulacastrum*, this buffering capacity may depend on rapid transcriptional activation of sulfate transporters that enables dynamic adjustment of sulfur pools under fluctuating salinity. Heavy metal stress revealed distinct spatial regulation of *SpSULTRs*. Cadmium treatment induced strong expression in roots, consistent with roots as the primary site of metal uptake and detoxification. This response likely supports enhanced synthesis of glutathione and related thiol compounds required for metal sequestration [55,56,57].

Copper stress produced different expression patterns between roots and leaves, indicating tissue-specific allocation of sulfur resources to meet localized detoxification demands. These patterns suggest that sulfate transporters contribute to systemic coordination of sulfur distribution under heterogeneous environmental stress [37,58,59,60]. These results support a model in which sulfate transporters in *S. portulacastrum* function as regulators of stress-responsive metabolic flux rather than simple nutrient transport components. By controlling sulfur entry into assimilation pathways, SULTRs influence the availability of metabolites required for redox regulation and detoxification [26,29,42,61]. Gene family remodeling combined with strong stress responsiveness suggests that sulfate transport systems have undergone evolutionary reorganization to support flexible metabolic adaptation to environmental challenges [38,62].

The SULTR3 subgroup differs from well-characterized sulfate uptake transporters in terms of its functional characteristics. While SULTR1 proteins mainly function in sulfate acquisition from the external environment, SULTR3 members are largely associated with sulfate distribution within cells and among tissues [40,42]. In Arabidopsis, AtSULTR3;1 is localized in chloroplasts and facilitates sulfate transport into plastids, providing sulfur substrates for assimilation and cysteine production [63]. Loss-of-function studies of *AtSULTR3s* have revealed disturbances in sulfur metabolism and altered responses to environmental stresses, indicating that proper intracellular sulfate allocation is important for maintaining the homeostasis of sulfur. In addition, AtSULTR3;5 is involved in sulfate movement through the root vasculature and contributes to root-to-shoot sulfate transport [39]. These findings suggest that different *SULTR3s* have acquired distinct functions in regulating sulfate partitioning according to tissue and cellular requirements. Evidence from rice further supports the functional diversification of the SULTR3 family. OsSULTR3;1 has been implicated in sulfate transport associated with selenium accumulation in grains, suggesting that SULTR3 proteins may influence the distribution of sulfur-related nutrients among different organs [64,65,66]. The distinct expression patterns of rice SULTR3 genes across tissues also indicate that individual members may function in specific developmental and physiological contexts. In *S. portulacastrum*, *SpSULTR3;1* and *SpSULTR3;2* showed pronounced responses to salt stress and were predominantly expressed in leaves. Although roots are the first site of salt perception and ion uptake, leaves are the central tissues for photosynthesis, sulfur assimilation, and the production of stress-related metabolites. The enhanced expression of leaf-expressed *SpSULTR3s* may reflect an increased demand for sulfate redistribution to support sulfur-dependent protective processes under saline conditions. Sulfur-derived metabolites, particularly cysteine and glutathione, are closely involved in ROS scavenging and redox regulation during salt stress. Therefore, the induction of *SpSULTR3;1* and *SpSULTR3;2* may facilitate stress adaptation by improving intracellular sulfur availability for the metabolism of antioxidants. The tissue-specific expression profiles of *SpSULTRs* further indicate that sulfate transport is precisely regulated according to cellular demand. Root-expressed members may contribute primarily to sulfate uptake and transport from the external environment, whereas *SULTR3s* expressed in leaves may participate in intracellular sulfate allocation and sulfur utilization during stress responses. Such division of labor may help *S. portulacastrum* maintain sulfur balance between growth and defense. Further functional studies using genetic modification and physiological analyses are required to determine the exact roles of *SpSULTR3;1* and *SpSULTR3;2* in sulfate transport and salt tolerance.

This study provides a framework for understanding how nutrient transport systems are reshaped during halophytic adaptation. Further functional analysis of individual SULTRs will be required to clarify their specific roles in sulfur partitioning and stress tolerance and to define how transporter-level regulation interacts with broader metabolic and signaling networks in extreme environments.

## 5. Conclusions

A total of 22 *SpSULTRs* were identified and grouped into three subfamilies based on phylogenetic relationships. Conserved domain analysis, gene structure comparison, and duplication analysis indicated that SpSULTRs underwent structural conservation and evolutionary diversification. Promoter and expression analyses further revealed that these genes are regulated by multiple environmental and developmental signals. Transcriptome profiling and qRT-PCR validation revealed that *SpSULTRs* exhibited distinct responses to salt, cadmium, and copper stress, with differences between roots and leaves. Of these, *SpSULTR3;1* and *SpSULTR3;2* showed prominent responses to salt stress and were mainly expressed in leaves. Their predicted interaction networks linked these proteins to sulfur assimilation, antioxidant metabolism, and stress-related pathways, suggesting that they may contribute to maintaining sulfur homeostasis during stress adaptation. Overall, this study expands our understanding of sulfate transport systems in halophytes and provides candidate genes for future investigations of sulfur-mediated stress tolerance. These findings establish a foundation for exploring how sulfate allocation and metabolism contribute to environmental adaptation in *S. portulacastrum* and other stress-resilient plants.

## Data Availability

All data analyzed can be found in the article.

## Supplementary Materials

The following supporting information can be downloaded at https://www.mdpi.com/article/10.3390/biology15161416/s1. Figure S1. The conserved motifs in SpSULTRs. Figure S2. Predicted 3D structure modeling of SpSULTRs. Figure S3. Co-expression network analysis. (A) Co-expression network analysis using SpSULTR3;1 as a bait. (B) Co-expression network analysis using SpSULTR3;2 as a bait. Figure S4. Relative transcription of *SpSULTRs*. Data represents mean ± SD (*n* = 3). The different letters above each bar indicate significant differences at *p* < 0.05 determined by one-way ANOVA analysis followed by Tukey’s multiple test. Figure S5. Relative transcription of *SpSULTRs*. Data represents mean ± SD (*n* = 3). The different letters above each bar indicate significant differences at *p* < 0.05 determined by one-way ANOVA analysis followed by Tukey’s multiple test. Table S1. Transcriptome data of *SpSULTRs* under cadmium treatment. Table S2. Primers used in this study. Table S3. Analysis of physicochemical properties of SpSULTRs. Table S4. Prediction of SpSULTRs secondary structure. Table S5. Ka, Ks, and Ka/Ks values for the homologous gene pair gene1 and gene2 in *SpSULTRs*. Table S6. WGD/segmental duplication event of *SpSULTRs*.
