# Supplementary material for: Transcriptome Reversal in Sulfate Transporter Involves Abiotic Stress in Sesuvium portulacastrum L
Source: Biology (Basel). 2026 Aug 18;15(16):1416. doi: 10.3390/biology15161416 (PMC13510048; doi:10.3390/biology15161416)
Supplement: Supplementary file 1 [file biology-15-01416-s001.zip › Supplement Tables.pdf]

Table S2. Primers used in this study.

Forward primer	Reverse primer	Description
TCTGTCGAGATTGGGCTTTTGA	TTTCGATTGCATGCTCCAACAG	qRT-PCR for *SpSULTR1;1*
TGTTGGTTCGATGACATCCTGT	TCAGCTAGCCATCTCAAAGTCC	qRT-PCR for *SpSULTR1;2*
AGTGAATTTCATGGCTGGTTGCT	CCCCGGATTTGCTAAAACAAGG	qRT-PCR for *SpSULTR1;3*
TCCATTGTCATATCTGCAGTGC	CTGATTTCGGGTAGCTTCTTCTC	qRT-PCR for *SpSULTR1;4*
CCCAAATGCCATACTTGCTTCC	AATCACCACTGGTCCTGGATTC	qRT-PCR for *SpSULTR1;5*
CCCAAATGCCATACTTGCTTCC	AATCACCACTGGTCCTGGATTC	qRT-PCR for *SpSULTR1;6*
TCAGTCTTTCTGGCCTTCCTTC	GAGGTGTGATGAATAGCAGGGT	qRT-PCR for *SpSULTR1;7*
ACTCCGCTATTCCATTACACCC	CTTCATCACTTCTCCTCCAGGG	qRT-PCR for *SpSULTR3;1*
TTGTTCCTCACTCCGCTATTCC	GCATGCTAATTCCACTCGTGTC	qRT-PCR for *SpSULTR3;2*
GGTGTTGTCTTTGAAGGTGTCG	CCTGGTGGGGAGATTGTCTTAC	qRT-PCR for *SpSULTR3;3*
GGTGTTGTCTTTGAAGGTGTCG	CCTGGTGGGGAGATTGTCTTAC	qRT-PCR for *SpSULTR3;4*
GTTGCAGGTCACAAATGTCCAA	AGTTGTCACTCCTCCCAATTCC	qRT-PCR for *SpSULTR3;5*
ACAGGGCCGTTTTCAAAATCAG	GACTCTTCATCTCGGATCCACC	qRT-PCR for *SpSULTR3;6*
GGCCATTCACCTTTGGAAAGTC	ATCTACAGGCCTCAACAGCATC	qRT-PCR for *SpSULTR3;7*
CCCAAACTGCACTTTCGAACAT	TCTTTCCAAGGTCTTCCTGAGC	qRT-PCR for *SpSULTR3;8*
CCCAAACTGCACTTTCGAACAT	TCTTTCCAAGGTCTTCCTGAGC	qRT-PCR for *SpSULTR3;9*
GTGCTCGTGACTCTTTTGTTCC	AGCTGCAGTGATCTTCTTTCCA	qRT-PCR for *SpSULTR3;10*
CAGTGCTTGTGACTCTCCTCTT	AATTCCACTTGTGTCGATTGCG	qRT-PCR for *SpSULTR3;11*
TGGTGTTCTCTTCATCTCCGTG	TCCAGTGGACAAATAAAGGCCA	qRT-PCR for *SpSULTR3;12*
ATGGGGCTGGTTGATTATGAGG	AGAGGACTTGGCGATTTGGATT	qRT-PCR for *SpSULTR4;1*
CCCTGGCACAACAATTTACAGG	CTACAGTTGCGTCCTCCTTTCT	qRT-PCR for *SpSULTR4;2*
AGGAGGCTATTCTATTGTGGCG	GGTGTTCTTGGAGGGTCGTATT	qRT-PCR for *SpSULTR4;3*
TTGGCATCGTTGAGGGTCT	CAGTGGGAACACGGAAAGC	qRT-PCR for *SpGAPDH*

Table S3. Analysis of physicochemical properties of SpSULTRs.

Gene name	Gene ID	Amino acids	Molecular weight (kDa)	pI	Aliphatic index	GRAVY	Subcellular localization
SpSULTR1;1	Spor0178440.1	649	71.12	8.64	109.28	0.367	Plasma membrane
SpSULTR1;2	Spor0498230.1	649	71.16	9.06	110.77	0.373	Plasma membrane
SpSULTR1;3	Spor0131040.1	591	64.68	9.11	107.46	0.402	Plasma membrane
SpSULTR1;4	Spor0006410.1	217	24.52	7.53	116.36	0.42	Plasma membrane
SpSULTR1;5	Spor0070770.1	664	72.87	9.12	111.16	0.37	Plasma membrane
SpSULTR1;6	Spor0268300.1	664	72.94	9.12	111.45	0.377	Plasma membrane
SpSULTR1;7	Spor0333730.1	665	72.98	9.12	111.88	0.381	Plasma membrane
SpSULTR3;1	Spor0489210.1	492	53.5	5.56	110.41	0.475	Plasma membrane
SpSULTR3;2	Spor0368690.1	658	72.32	8.72	103.89	0.389	Plasma membrane
SpSULTR3;3	Spor0549240.1	649	71.57	9.1	108.15	0.4	Plasma membrane
SpSULTR3;4	Spor0196340.1	649	71.54	8.91	108.75	0.406	Plasma membrane
SpSULTR3;5	Spor0499930.1	651	72.43	8.87	109.17	0.344	Plasma membrane
SpSULTR3;6	Spor0176650.1	651	72.57	8.88	108.57	0.331	Plasma membrane
SpSULTR3;7	Spor0129190.1	593	65.4	8.76	107.52	0.343	Plasma membrane
SpSULTR3;8	Spor0143430.1	679	74.39	9.12	112.31	0.418	Plasma membrane
SpSULTR3;9	Spor0474850.1	679	74.45	9.24	112.74	0.431	Plasma membrane
SpSULTR3;10	Spor0353760.1	686	75.24	9.06	110.6	0.368	Plasma membrane
SpSULTR3;11	Spor0176660.1	531	57.84	9.18	119.53	0.499	Plasma membrane
SpSULTR3;12	Spor0129200.1	532	57.61	8.96	118.95	0.63	Plasma membrane
SpSULTR4;1	Spor0551490.1	698	76.06	9.01	108.09	0.352	Plasma membrane
SpSULTR4;2	Spor0410470.1	698	75.97	9.06	109.21	0.375	Plasma membrane
SpSULTR4;3	Spor0194090.1	641	69.42	9.09	107.54	0.371	Plasma membrane

Table S4. Prediction of SpSULTRs secondary structure.

Protein	Alpha helix (%)	Extended strand (%)	Random coil (%)	Distribution of secondary structure elements
SpSULTR1;1	50.85	10.94	38.21	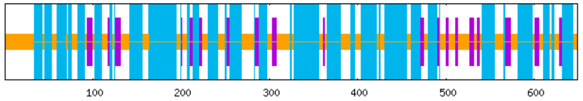
SpSULTR1;2	49.46	10.79	39.75	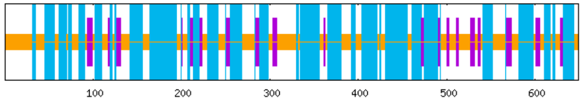
SpSULTR1;3	48.39	11.17	40.44	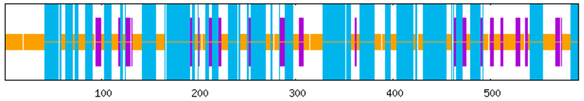
SpSULTR1;4	51.15	15.21	33.64	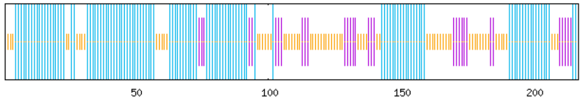
SpSULTR1;5	48.49	10.54	40.96	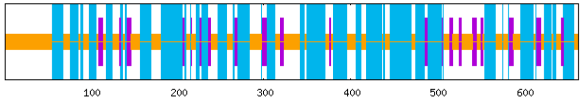
SpSULTR1;6	48.19	10.84	40.96	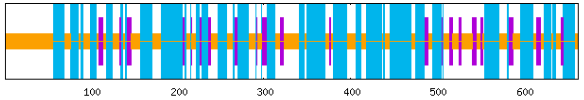
SpSULTR1;7	49.32	10.68	40	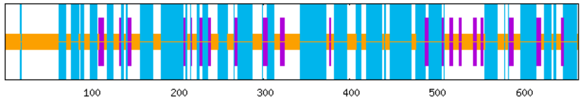
SpSULTR3;1	56.71	9.15	34.15	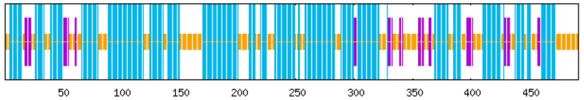
SpSULTR3;2	49.24	10.79	39.97	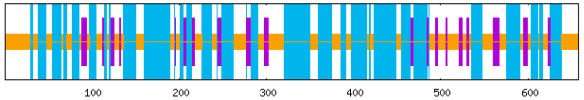

SpSULTR3;3	50.08	11.56	38.37	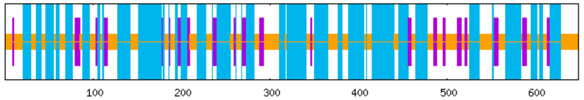
SpSULTR3;4	49.15	11.4	39.45	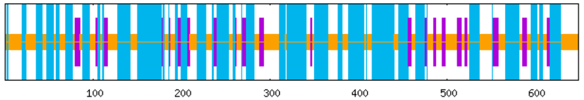
SpSULTR3;5	49.46	10.91	39.63	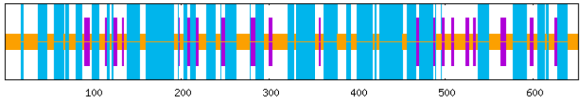
SpSULTR3;6	50.84	10.29	38.86	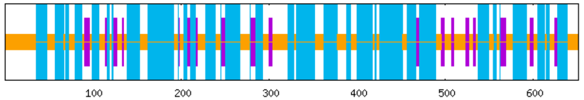
SpSULTR3;7	51.77	9.95	38.28	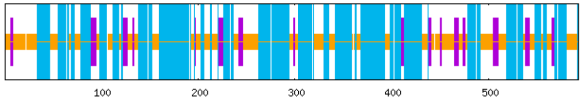
SpSULTR3;8	47.28	11.05	41.68	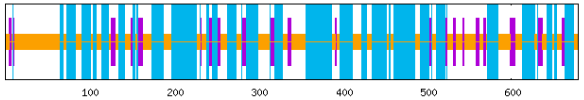
SpSULTR3;9	48.75	10.9	40.35	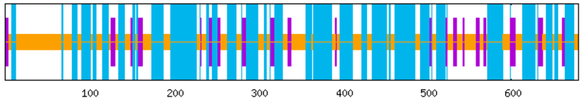
SpSULTR3;10	47.96	11.22	40.82	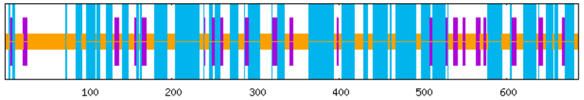
SpSULTR3;11	51.41	12.24	36.35	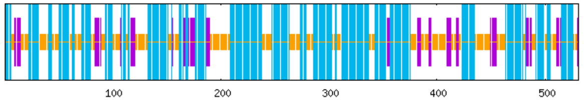
SpSULTR3;12	53.57	12.03	34.4	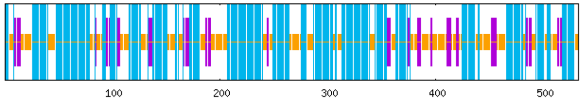

SpSULTR4;1	46.7	10.74	42.55	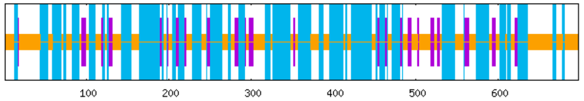
SpSULTR4;2	46.85	10.32	42.84	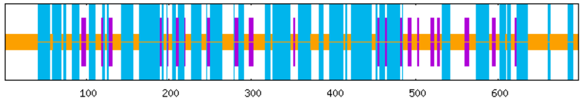
SpSULTR4;3	45.87	9.2	44.93	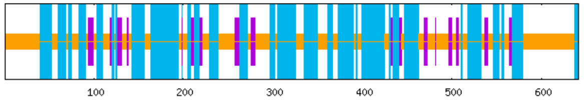

Table S5. Ka, Ks, and Ka/Ks values for the homologous gene pair gene1 and gene2 in *SpSULTRs*.

Gene1	Gene2	Ka	Ks	Ka/Ks
SpSULTR1;1	*SpSULTR1;2*	0.012935023	0.048824779	0.264927428
SpSULTR1;3	*SpSULTR1;1*	0.027643904	0.07780701	0.355288092
SpSULTR1;3	*SpSULTR1;2*	0.028444509	0.07235262	0.393137236
SpSULTR1;6	*SpSULTR1;5*	0.011389232	0.082214794	0.1385302
SpSULTR1;6	*SpSULTR1;7*	0.007354639	0.077488211	0.094913004
SpSULTR1;7	*SpSULTR1;5*	0.00533632	0.055324613	0.096454728
SpSULTR3;2	*SpSULTR3;1*	0.053277286	0.156791655	0.339796696
SpSULTR3;3	*SpSULTR3;2*	0.255202889	2.640809783	0.096638119
SpSULTR3;3	*SpSULTR3;1*	0.279517248	2.503222269	0.111662976
SpSULTR3;4	*SpSULTR3;3*	0.003391561	0.043864134	0.077319679
SpSULTR3;4	*SpSULTR3;2*	0.255220006	2.39269668	0.10666626
SpSULTR3;4	*SpSULTR3;1*	0.279542414	2.355156736	0.118693762
SpSULTR3;6	*SpSULTR3;5*	0.006694605	0.045546149	0.146985095
SpSULTR3;7	*SpSULTR3;6*	0.010343081	0.060019513	0.172328636
SpSULTR3;7	*SpSULTR3;5*	0.00663395	0.062588424	0.10599324
SpSULTR3;8	*SpSULTR3;12*	0.177468909	2.38165041	0.074515096
SpSULTR3;8	*SpSULTR3;9*	0.005876941	0.034824484	0.168758896
SpSULTR3;9	*SpSULTR3;12*	0.178725491	2.429876484	0.073553324
SpSULTR3;10	*SpSULTR3;8*	0.01046909	0.082710844	0.126574581
SpSULTR3;10	*SpSULTR3;9*	0.010474231	0.087069552	0.120297284
SpSULTR3;10	*SpSULTR3;12*	0.176825398	2.512112209	0.070389132
SpSULTR3;12	*SpSULTR3;11*	0.016937911	0.075639074	0.223930706
SpSULTR4;2	*SpSULTR4;1*	0.008058528	0.086148006	0.093542829
SpSULTR4;3	*SpSULTR4;2*	0.008450794	0.065396286	0.129224366
SpSULTR4;3	*SpSULTR4;1*	0.004560351	0.029983317	0.152096273

Table S6. WGD/segmental duplication event of *SpSULTRs*.

Gene name	Duplication type
SpSULTR1;1	WGD or Segmental
SpSULTR1;2	WGD or Segmental
SpSULTR1;3	WGD or Segmental
SpSULTR1;4	WGD or Segmental
SpSULTR1;5	WGD or Segmental
SpSULTR1;6	WGD or Segmental
SpSULTR1;7	WGD or Segmental
SpSULTR3;1	WGD or Segmental
SpSULTR3;2	WGD or Segmental
SpSULTR3;3	WGD or Segmental
SpSULTR3;4	WGD or Segmental
SpSULTR3;5	WGD or Segmental
SpSULTR3;6	WGD or Segmental
SpSULTR3;7	WGD or Segmental
SpSULTR3;8	WGD or Segmental
SpSULTR3;9	WGD or Segmental
SpSULTR3;10	WGD or Segmental
SpSULTR3;11	WGD or Segmental
SpSULTR3;12	WGD or Segmental
SpSULTR4;1	WGD or Segmental
SpSULTR4;2	WGD or Segmental
SpSULTR4;3	WGD or Segmental
